# Unmet needs in the treatment of idiopathic pulmonary fibrosis―insights from patient chart review in five European countries

**DOI:** 10.1186/s12890-017-0468-5

**Published:** 2017-09-15

**Authors:** Toby M. Maher, Maria Molina-Molina, Anne-Marie Russell, Francesco Bonella, Stéphane Jouneau, Elena Ripamonti, Judit Axmann, Carlo Vancheri

**Affiliations:** 1 NIHR Respiratory Biomedical Research Unit, Royal Brompton Hospital and Fibrosis Research Group, National Heart and Lung Institute, Imperial College London, London, UK; 2 University Hospital of Bellvitge, Institut d’Investigacions Biomèdiques de Bellvitge (IDIBELL), Barcelona, and Centro de Investigación Biomédica en Red Enfermedades Respiratorias (CIBERES), Barcelona, Spain; 3 Ruhrlandklinik, University Hospital Essen, Essen, Germany; 4 Hôpital Pontchaillou, IRSET UMR 1085, Université de Rennes 1, Rennes, France; 5 Elma Research S.R.L, Milan, Italy; 6 F. Hoffmann-La Roche Ltd., Basel, Switzerland; 7 Regional Referral Centre for Rare Lung Diseases, University of Catania, Catania, Italy; 8 Fibrosis Research Group, Inflammation, Repair and Development Section, NHLI, Sir Alexander Fleming Building, Imperial College London, London, SW7 2AZ UK

**Keywords:** Antifibrotics, Idiopathic pulmonary fibrosis, Questionnaire, Treatment patterns, Unmet needs

## Abstract

**Background:**

Two antifibrotic drugs, pirfenidone and nintedanib, are approved by the European Medicines Agency and the US Food and Drug Administration for the treatment of idiopathic pulmonary fibrosis (IPF). In this analysis, treatment patterns of European patients with IPF were investigated to understand antifibrotic prescribing and identify unmet needs in IPF treatment practice.

**Methods:**

Between February and March 2016, respiratory physicians from France, Germany, Italy, Spain, and the UK participated in an online questionnaire designed to collect information on IPF treatment patterns in patients under their care. Patients were categorized as treated (received approved antifibrotics) or untreated (did not receive approved antifibrotics, but may have received other unapproved therapies). Classification of IPF diagnosis (confirmed/suspected) and severity (‘mild’/‘moderate’/‘severe’) for each patient was based on the individual physician’s report. Patients’ perspectives were not recorded in this study.

**Results:**

In total, 290 physicians responded to the questionnaire. Overall, 54% of patients with IPF did not receive treatment with an approved antifibrotic. More patients had a confirmed IPF diagnosis in the treated (84%) versus the untreated (51%) population. Of patients with a confirmed diagnosis, 40% did not receive treatment. The treated population was younger than the untreated population (67 vs 70 years, respectively; *p* ≤ 0.01), with more frequent multidisciplinary team evaluation (83% vs 57%, respectively; *p* ≤ 0.01). A higher proportion of untreated patients had forced vital capacity > 80% at diagnosis versus treated patients. Of patients with ‘mild’ IPF, 71% did not receive an approved antifibrotic versus 41% and 60% of patients with ‘moderate’ and ‘severe’ IPF, respectively.

**Conclusions:**

Despite the availability of antifibrotic therapies, many European patients with confirmed IPF do not receive approved antifibrotic treatment. Importantly, there appears to be a reluctance to treat patients with ‘mild’ or ‘stable’ disease, and instead adopt a ‘watch and wait’ approach. More education is required to address diagnostic uncertainty, poor understanding of IPF and its treatments, and issues of treatment access. There is a need to increase physician awareness of the benefits associated with antifibrotic treatment across the spectrum of IPF severity.

**Electronic supplementary material:**

The online version of this article (10.1186/s12890-017-0468-5) contains supplementary material, which is available to authorized users.

## Background

Idiopathic pulmonary fibrosis (IPF) is a chronic, debilitating, irreversible, and progressive lung disease characterized by exertional dyspnea and cough [1, 2]. Patients with IPF have a poor prognosis, with median survival following diagnosis previously reported as lower than that for many common types of cancer at between 2 and 5 years [1–8].

The reported incidence of IPF has been estimated to range from 2.8 to 9.3 cases per 100,000 population per year, in Europe and North America [9]. The prevalence of IPF in Europe is thought to range from 1.25 to 23.4 cases per 100,000 population [10]. There is evidence that the incidence, prevalence, and number of deaths from IPF may be increasing [9, 11–13].

Two antifibrotic drugs, pirfenidone and nintedanib, are approved by the European Medicines Agency and the US Food and Drug Administration for the treatment of IPF, and both are recommended in international treatment guidelines [14]. In the Phase III ASCEND and CAPACITY trials, pirfenidone significantly reduced the risk of disease progression or death compared with placebo [15, 16]. In the Phase III INPULSIS trials, nintedanib reduced the risk of disease progression versus placebo in patients with IPF [17].

Following the approval and recommendation of pirfenidone and nintedanib for the treatment of IPF, we conducted a patient chart audit using an online physician survey to investigate pharmacological treatment patterns, understand antifibrotic prescribing, and identify unmet needs in IPF treatment practice in Europe.

## Methods

### Study design and patients

This was a patient chart audit survey involving respiratory physicians from France, Germany, Italy, Spain, and the UK. Between February and March 2016, physicians participated in an online questionnaire (35–40 min) designed to collect information on IPF treatment patterns. The questionnaire was developed by Elma Research, an independent market research agency, on behalf of F. Hoffmann-La Roche Ltd. The questionnaire was available in English, French, German, Italian, and Spanish; all responses were precoded as numbers so translation was not required. Patients’ perspectives were not recorded in this study.

Responses were collected from physicians who had consulted with at least six (France, Italy, Spain) or 10 (Germany, UK) patients with IPF within the previous 3 months. The number of patients with IPF required for each physician varied by country to account for inter-country differences in patient population size. Italian and British physicians were selected from a list of panelists held by Elma Research, which includes physicians willing to take part in market research. In France, Germany, and Spain, external suppliers invited the physicians to participate on behalf of Elma Research. Physicians were asked to report on the last six patients (eight in the UK) with IPF they saw, regardless of any specific diagnostic or therapeutic features. No patient-identifiable data were collected and patients remained anonymous. All respondents received a cash incentive, which was awarded for participation in the research; i.e., this was not on a per-patient basis. Respondents agreed to complete the form personally, i.e., not to delegate the form completion to another staff member.

Patients were categorized as being in one of the following populations based on their last consultation:

- Treated population―those patients who had received approved antifibrotics for the treatment of IPF
- Untreated population―those patients who had not received approved antifibrotics.

In both the treated and untreated populations, patients may have been receiving concomitant therapies, such as N-acetylcysteine (NAC), steroids (prednisolone), immunosuppressants, and/or oxygen. Patients may also have received pharmacological therapies for the palliation of symptoms associated with IPF, and therapies for concomitant conditions. The retrospective nature of the survey meant that participation did not prompt any change in patient care; concomitant therapies may have been continuing or newly initiated at the last visit at the discretion of the treating physician. It is possible that continuing therapies may have been established in accordance with superseded clinical guidelines [2, 18].

#### Assessments

The questionnaire assessed a number of factors, including baseline demographics, IPF diagnosis, disease severity, treatments, and comorbidities (Additional files 1 and 2).

Pulmonary function and exercise capacity (6-min walk distance [6MWD]) were recorded from diagnosis and from the most recent consultation, where these data were available. Classification of IPF diagnosis (confirmed/suspected), severity (‘mild’/‘moderate’/‘severe’), and evolution of severity (improvement/stable/worsening) for each patient was based on the individual physician’s report, i.e., no pre-defined forced vital capacity (FVC) or carbon monoxide diffusing capacity (DLco) threshold was given to determine disease severity, and individual physicians may have applied different thresholds. Physicians were asked to report the number of acute exacerbations of IPF that resulted in hospitalization or an emergency room visit within the last year; acute exacerbations were defined according to clinical presentation and no standard criteria or adjudication were applied.

#### Statistical analysis

Statistical analyses were performed by a senior data analyst from Elma Research in April 2016 using Quantum v 5.8, once all questionnaires were completed and information on the number and percentage of respondents per answer were summarized. Comparisons between treated and untreated populations were performed using t-tests.

In the UK and Italy, expert centers were classified as those authorized to prescribe pirfenidone, while in France, Germany, and Spain, expert centers were defined as:

- General university hospital with > 60 patients in care and multidisciplinary team (MDT) available
- OR, office-based physicians who have an MDT available and care for > 60 patients
- OR, working in a lung clinic and have an MDT.

Centers not meeting these criteria, or who were not authorized to prescribe pirfenidone in the UK and Italy, were classed as non-expert centers. A subgroup analysis comparing expert and non-expert centers was performed for the following endpoints: IPF diagnosis, time until next consultation, goals with current treatment, and frequency of acute exacerbations. Comparisons between expert and non-expert subgroups were performed using t-tests.

Data for the number of physicians based at centers in the UK authorized to prescribe pirfenidone were weighted equally for prescribing and non-prescribing centers (50% each). In Italy, data were weighted 67% and 33% for prescribing and non-prescribing centers. To avoid duplication of data from patients treated with pirfenidone in Italy or in the UK, patients reported by non-prescribing centers as co-managed with an authorized prescribing center within the last 3 months were not included in the analysis.

## Results

### Physicians

Overall, there were 290 respondent physicians from Germany, France, Italy, the UK, and Spain reporting on 1838 patients. Out of 119 physicians from the UK and Italy, 80 (67.2%) were working in expert centers, which by definition were authorized to prescribe pirfenidone (Table 1). In France, Germany, and Spain, a total of 90/171 (52.6%) physicians were designated as being from expert centers. MDT evaluation, which alone did not confer status as an expert center, was available in the centers of 213 (73.4%) physicians (Table 1).

**Table 1:** Physician characteristics

Factor, *n* (%)	Physicians *N* = 290
Country^a^	
Germany	60 (20.7)
France	51 (17.6)
Italy	70 (24.1)
United Kingdom (UK)	49 (16.9)
Spain	60 (20.7)
Type of practice	
General hospital	157 (54.1)
Centre specializing in lung diseases	120 (41.4)
Office-based practice	42 (14.5)
MDT available	213 (73.4)
MDT team members	
Respiratory specialist/pulmonologist	212 (99.5)
Radiologist	206 (96.7)
Pathologist	165 (77.5)
ILD/IPF Specialist Nurse	70 (32.9)
Other	44 (20.7)
Expert center (France, Germany, and Spain)^a^	90/171 (52.6)
General University Hospital with >60 patients and MDT^b^	40 (23.4)
Office-based physicians with >60 patients and MDT	5 (2.9)
Lung clinic with MDT available^b^	47 (27.5)
Expert center (UK and Italy)^a^	
Authorized to prescribe pirfenidone	80/119 (67.2)

For individual questions asked, please refer to Additional files 1 and 2

*MDT* multidisciplinary team, *ILD* interstitial lung disease, *IPF*, idiopathic pulmonary fibrosis

^a^Unweighted data

^b^Two centers qualified under both criteria

### Patients

**Fig. 1:**
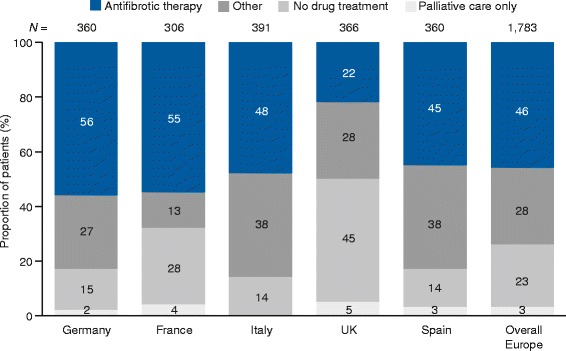
Proportion of patients that are treated or untreated across European countries. Unweighted data. For individual questions asked, please refer to Additional files 1 and 2

Of the 1838 patients, 55 patients in Italy and the UK were co-managed with a prescribing center and were therefore excluded from further analysis to avoid double-counting. Of the remaining 1783 patients analyzed, 955 (53.6%) did not receive treatment with either approved antifibrotic drug (Fig. 1). The proportions of patients starting a new medication, switching to a different medication, or discontinuing a medication at their last consultation were 18.5, 6.8, and 1.2%, respectively.

**Fig. 2:**
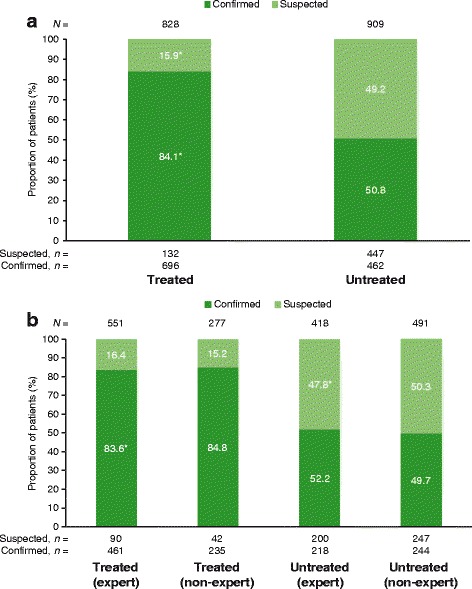
Type of diagnosis, (**a**) pooled population (**b**) expert versus non-expert centers ^*^ *p* ≤ 0.01 for (**a**) treated population versus untreated population and (**b**) expert population versus non-expert population. Excluding patients receiving only palliative care. Number of patients with a confirmed diagnosis at expert (691/993, 69.6%) versus non-expert centers (494/790, 62.5%)―*p* ≤ 0.01. Number of patients with confirmed IPF treated at expert (461/679, 67.9%) versus non-expert centers (235/479, 49.2%)―*p* ≤ 0.01. For individual questions asked, please refer to Additional files 1 and 2. *IPF* idiopathic pulmonary fibrosis

Excluding patients who received palliative care only or palliative care only + oxygen therapy (*N* = 46), 828 patients (47.7%) received antifibrotic treatment and 909 patients (52.3%) did not receive antifibrotic treatment. More patients in the treated population had a confirmed diagnosis of IPF versus patients in the untreated population (Fig. 2a). Of 1158 patients with a confirmed IPF diagnosis, 462 (39.9%) did not receive treatment with an approved antifibrotic drug. More patients at expert centers had a confirmed diagnosis of IPF than at non-expert centers (70.1% vs 62.4%, respectively) (Fig. 2b); of patients with a confirmed diagnosis of IPF, antifibrotic treatment was received by 67.9% (461/679) of those at expert centers compared with 49.1% (235/479) of those at non-expert centers (Fig. 2b).

The treated population was younger than the untreated population and had more frequent MDT evaluation. Treated patients generally had a lower proportion of lung and cardiovascular comorbidities compared with untreated patients (Table 2). Significantly more treated patients were candidates for lung transplantation compared with untreated patients (Table 2).

**Table 2:** Patient characteristics

Factor, mean (SD) or *n* (%)	Treated (*N* = 828)	Untreated (*N* = 909)
Mean (SD) age, years	66.6 (9.3)	70.1 (11.4)^**^
Male	568 (68.6)	570 (62.7)^**^
MDT evaluation	687 (83.0)	520 (57.2)^**^
Confirmed IPF	696 (84.1)	462 (50.8)^**^
IPF severity at diagnosis		
Mild	213 (25.7)	395 (43.5)^**^
Moderate	530 (64.0)	367 (40.4)^**^
Severe	85 (10.3)	147 (16.2)^**^
Mean (SD) time from diagnosis to most recent consultation, months	15.8 (21.8)	15.9 (22.4)
Symptomatic at initiation of current treatment	746 (90.1)	430 (85.4)^a**^
Candidate for lung transplantation	154 (18.6)	66 (7.3)^**^
Lung comorbidities	323 (39.0)	460 (50.6)^**^
Emphysema	187 (22.6)	299 (32.9)^**^
Lung cancer	20 (2.4)	46 (5.1)^**^
Pulmonary hypertension	184 (22.2)	229 (25.2)
CV comorbidities	320 (38.6)	406 (44.7)^*^
High risk of coronary artery disease	131 (15.8)	153 (16.8)
Coronary artery disease without history of MI or stroke	119 (14.4)	143 (15.7)
Coronary artery disease with history of MI	88 (10.6)	135 (14.9)^**^
Other comorbidities		
GERD	262 (31.6)	257 (28.3)
Depression	199 (24.0)	200 (22.0)
Obstructive sleep apnea syndrome	103 (12.4)	109 (12.0)
Increased risk of bleeding^b^	38 (4.6)	44 (4.8)
Other	111 (13.4)	149 (16.4)

*p* values represent treated population versus untreated population. ^*^ *p* ≤ 0.05; ^**^ *p* ≤ 0.01

^a^ *N* = 504, patients who received no treatment were excluded

^b^e.g., due to use of anticoagulation therapy or concomitant diseases

For individual questions asked, please refer to Additional file 2

*CV* cardiovascular, *GERD* gastroesophageal reflux disease, *IPF* idiopathic pulmonary fibrosis, *MDT* multidisciplinary team, *MI* myocardial infarction, *SD* standard deviation

A total of 1435 patients had data available regarding non-antifibrotic therapies that were being prescribed for IPF at the time of questionnaire completion (this information was not collected in France). Of 638 treated patients, the following patients also received another therapy: NAC = 88 (13.8%), steroids = 60 (9.4%), immunosuppressants = 11 (1.7%), palliative care = 3 (0.5%), oxygen = 172 (27.0%), and other pharmacological therapy = 164 (25.7%). In the untreated population (*n* = 797), the following patients received: NAC = 219 (27.5%), steroids = 251 (31.5%), immunosuppressants = 62 (7.8%), palliative care = 101 (12.7%), oxygen = 300 (37.6%), and other pharmacological therapy = 269 (33.8%).

Among untreated patients (including patients in France), 405 patients (45%) were reported as receiving ‘no drug treatment’. Some of these patients were also reported as receiving oxygen therapy (61 [15.1%]), other therapy (pharmacological or non-pharmacological; 37 [9.1%]), or palliative care including morphine (13 [3.2%]). Untreated patients receiving palliative care only were older (mean age = 81 years) than those receiving no pharmacological treatment (71 years), oxygen therapy (74 years), or other therapy (73 years). The most common reasons given for why the patient was not receiving any drug treatment were: lack of, or few, symptoms related to IPF (27%), stable disease (26%), old age (20%), and physician-reported good quality of life (20%).

The three most common treatment goals reported by physicians in both the treated and untreated populations were to prolong survival/reduce risk of mortality, improve quality of life, and stabilize disease (Table 3). To prolong survival/reduce risk of mortality was the most important treatment goal across all groups in the expert versus non-expert analyses, with the exception of the untreated non-expert population, where improvement in quality of life was the most important treatment goal (Table 3).

**Table 3:** Most important treatment goals with current treatment

Goal, *n* (%)	Pooled population		Treated		Untreated	
	Treated *N* = 828	Untreated *N* = 405	Expert *N* = 551	Non-expert *N* = 277	Expert *N* = 176	Non-expert *N* = 229
Prolong survival/reduce risk of mortality	402 (48.6)	174 (43.0)	273 (49.5)	129 (46.6)	80 (45.5)	94 (41.0)
Improvement of quality of life	314 (37.9)	179 (44.2)^*^	194 (35.2)	120 (43.3)	75 (42.6)	104 (45.4)
Overall disease stabilization	334 (40.3)	152 (37.5)	234 (42.5)	100 (36.1)	64 (36.4)	88 (38.4)
Stabilization of predicted% FVC	279 (33.7)	56 (13.8)^**^	176 (32.0)	102 (36.8)	23 (13.1)	33 (14.4)
Stabilization of quality of life	248 (30.0)	135 (33.3)	164 (29.8)	83 (30.0)	70 (39.8)	65 (28.4)
Improvement of symptoms	236 (28.5)	142 (35.1)^*^	161 (29.2)	76 (27.4)	57 (32.4)	85 (37.1)
Stabilization of symptoms	215 (26.0)	143 (35.3)^**^	135 (24.5)	81 (29.2)	68 (38.6)	75 (32.8)
Decrease in number of exacerbations	208 (25.1)	94 (23.2)	153 (27.8)	55 (19.9)	40 (22.7)	53 (23.1)
Avoid pulmonary hospitalizations	156 (18.8)	122 (30.1)^**^	101 (18.3)	55 (19.9)	46 (26.1)	76 (33.2)
Stabilization of predicted% DLco	92 (11.1)	19 (4.7)^**^	61 (11.1)	31 (11.2)	3 (1.7)	15 (6.6)

*DLco* carbon monoxide diffusing capacity; *FVC* forced vital capacity

Respondents were asked to pick the three most important goals; for individual questions asked, please refer to Additional file 2

*p* values represent treated population versus untreated population. ^*^ *p* ≤ 0.05; ^**^ *p* ≤ 0.01

A larger proportion of the treated population (84.9%) had ≤ 3 months until their next consultation versus the untreated population (59.3%) (Additional file 3).

### Disease severity and pulmonary function

**Fig. 3:**
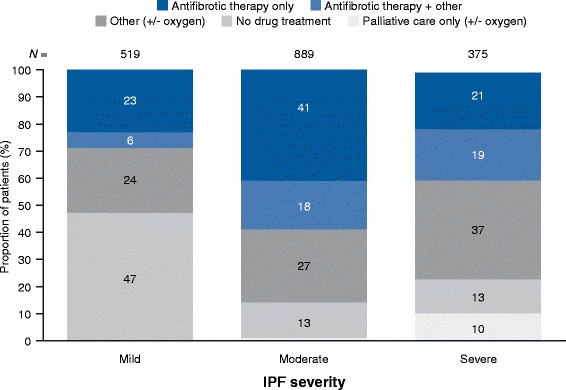
Overall proportion of treated and untreated patients based on current disease severity Classification of disease severity was based on the subjective determination of individual physicians for each patient. For individual questions asked, please refer to Additional files 1 and 2. *IPF* idiopathic pulmonary fibrosis

Of 519 patients with ‘mild’ IPF, 71% (*n* = 370) did not receive treatment with an approved antifibrotic compared with 41% (*n*/*N* = 361/889) and 60% (*n*/*N* = 224/375) of patients with ‘moderate’ and ‘severe’ IPF, respectively (Fig. 3). The proportion of patients with ‘mild’ IPF who did not receive treatment with an approved antifibrotic was 40.4, 41.4, 37.5, 51.4, and 44.7% in Germany, France, Italy, the UK, and Spain, respectively.

In addition, a higher proportion of untreated versus treated patients had FVC > 80% or did not have their pulmonary function assessed at diagnosis (Table 4). At the most recent consultation, significantly more untreated patients versus treated patients had ‘mild’ (40.7% vs 18.0%; *p* ≤ 0.01) and/or stable (50.9% vs 31.3%; *p* ≤ 0.01) IPF (Table 4). Likewise, more untreated patients had FVC > 80% at last check-up than treated patients; however, fewer untreated patients had an FVC measurement at their most recent check-up compared with treated patients (Table 4).

**Table 4:** Disease characteristics

Factor, *n* (%)	Treated *N* = 828	Untreated *N* = 909
Diagnostic values		
FVC		
FVC > 80%	94 (11.4)	163 (17.9)^**^
FVC 71–80%	143 (17.3)	183 (20.1)
FVC 50–70%	425 (51.3)	335 (36.9)^**^
FVC < 50%	102 (12.3)	122 (13.4)
FVC not tested	64 (7.7)	106 (11.7)^**^
DLco		
DLco < 35%	74 (8.9)	85 (9.4)
DLco ≥ 35%	634 (76.6)	612 (67.3)^**^
DLco not tested	121 (14.6)	212 (23.3)^**^
6MWD		
6MWD < 150 m	71 (8.6)	71 (7.8)
6MWD ≥ 150 m	460 (55.6)	323 (35.5)^**^
Not tested	297 (35.9)	516 (56.8)^**^
Last visit		
Mild IPF (current level)	149 (18.0)	370 (40.7)^**^
FVC		
FVC > 80%	39 (4.7)	119 (13.1)^**^
FVC 71–80%	116 (14.0)	134 (14.7)
FVC 50–70%	427 (51.6)	279 (30.7)^**^
FVC < 50%	148 (17.9)	141 (15.5)
FVC not tested	98 (11.8)	236 (26.0)^**^
Evolution in severity level^a^ from diagnosis to last check-up		
Improvement	38 (4.6)	36 (4.0)
Stable	637 (76.9)	782 (86.0)^**^
Worsening	154 (18.6)	92 (10.1)^**^
Type of progression		
Stable IPF	259 (31.3)	463 (50.9)^**^
Slow progressing	383 (46.3)	291 (32.0)^**^
Progressive	159 (19.2)	114 (12.5)^**^
Fast progressing	27 (3.3)	41 (4.5)

For individual questions asked, please refer to Additional file 2

*6MWD* 6-min walk distance, *DLco* carbon monoxide diffusing capacity, *FVC* forced vital capacity, *IPF* idiopathic pulmonary fibrosis

^a^Evolution in severity levels was defined as follows: Improvement = from Moderate to Mild/from Severe to Moderate or Mild; Stable = unchanged level of severity at diagnosis to last check-up; Worsening = from Mild to Moderate or Severe/from Moderate to Severe

*p* values represent treated population versus untreated population. ^*^ *p* ≤ 0.05; ^**^ *p* ≤ 0.01

**Fig. 4:**
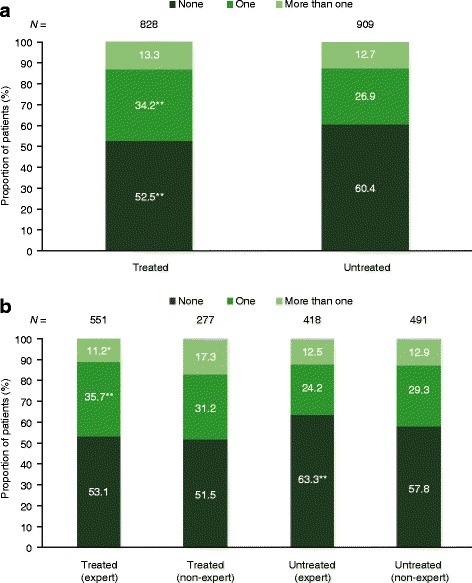
Exacerbations in the last year, (**a**) pooled population (**b**) expert versus non-expert centers. ^*^ *p* ≤ 0.05; ^**^ *p* ≤ 0.01 for (**a**) treated population versus untreated population and (**b**) expert population versus non-expert population. Excluding patients receiving only palliative care. For individual questions asked, please refer to Additional file 1 and Fig. 2

According to physician responses, a high proportion of patients had experienced an acute exacerbation of IPF in the year before completion of the survey, resulting in hospitalization or an emergency room visit (treated = 47.5% vs untreated = 39.6%) (Fig. 4a). A total of 16% of patients with ‘mild’ IPF had a physician-reported acute exacerbation compared with 38% and 32% of patients with ‘moderate’ and ‘severe’ IPF, respectively. Patients at non-expert centers had slightly more acute exacerbations than patients at expert centers, whether they were treated or untreated (Fig. 4b).

## Discussion

Our results show that approximately 40% of European patients with confirmed IPF do not receive antifibrotic treatment despite the regulatory approval of two antifibrotic therapies and the recommendation in international guidelines that the majority of individuals with IPF should be offered antifibrotic treatment. Indeed, at the time of the survey, antifibrotic therapy had been available for at least 2 years in all the countries surveyed, and it is therefore important to consider reasons for the observed treatment pattern.

Treatment requires a confident diagnosis of IPF, and it may be that a lack of awareness about IPF as a potential diagnosis and/or a lack of referral to specialist centers for MDT diagnostic assessment have an impact upon treatment practices. Our results show that a higher proportion of untreated patients had suspected IPF than treated patients and a lower proportion of untreated patients had an MDT evaluation at diagnosis. Uncertain diagnosis is also a key barrier to treatment in patients with suspected IPF, which will potentially be addressed by two clinical trials currently investigating the efficacy of antifibrotics in non-IPF interstitial lung diseases (NCT03099187 and NCT02999178).

In our analysis of treatment patterns in expert versus non-expert centers, more patients had a confirmed diagnosis of IPF at expert centers. In addition, a higher proportion of patients in the untreated population did not have an FVC (12% vs 8%), DLco (23% vs 15%), or 6MWD (57% vs 36%) measurement at baseline compared with the treated population. These differences between the untreated and treated populations could reflect a number of issues, including difficulty with interpreting dynamic changes in pulmonary function or reduced monitoring in patients considered to be unsuitable for treatment by their physician.

Previous studies have shown that patients often visit several healthcare professionals before being diagnosed with IPF, with the process of obtaining a confirmed diagnosis taking in excess of 1 year in the majority of cases [19, 20]. Misdiagnosis and a lack of knowledge about IPF in primary care are cited as key reasons for delayed referral to specialist centers [20, 21]. Our data suggest that referral to a non-specialist pulmonologist may be another barrier to diagnosis and treatment access. Patients in several areas across the EU have reported limited access to a full MDT to facilitate diagnosis [21]. Once a diagnosis has been made, areas of unmet needs include a lack of awareness of available approved antifibrotic therapy and/or information and resources on pulmonary fibrosis from both a patient and a healthcare professional perspective [22–24]. Indeed, in our experience, informed patients with a good knowledge of their condition and the available treatments are more likely to request referral to specialist care and/or access to treatment than those with less knowledge about their condition. Similar factors were highlighted in the European IPF Patient Charter [21] and may contribute to the delayed diagnosis and treatment of IPF.

Our results indicate that many patients with IPF that is perceived to be ‘mild’ or ‘stable’ by their physician were not treated with an antifibrotic, suggesting physicians were adopting a ‘watch and wait’ approach. Indeed, a large group of patients who did not receive an antifibrotic received no treatment at all, with the most common reasons for this being lack of symptoms and/or lack of disease progression. The data gathered in this survey suggest that treated patients had more severe disease than untreated patients: they were more likely to have an FVC < 70% at diagnosis and follow-up, they were more likely to have disease rated as ‘moderate’ by their physician, they tended to have more acute exacerbations than untreated patients, and they were more likely to be candidates for lung transplantation (although it should be acknowledged that this may have been because they were younger and/or had fewer comorbidities than untreated patients, rather than reflecting more severe disease).

One possible explanation for patients with ‘mild’ or ‘stable’ IPF remaining untreated is a lack of physician confidence in the evidence base. The limitations of our survey design prevented further investigation of this possibility; however, a survey of respiratory physicians has previously reported that physicians who waited for disease progression before initiating antifibrotic therapy were less likely to agree that antifibrotics can significantly slow disease progression compared with physicians who treated within 4 months of diagnosis [25]. However, the available evidence increasingly points toward early intervention in this progressive, unpredictable, irreversible, and fatal disease [1, 26–31], especially as experience from other lung diseases suggests that physicians tend to underestimate the severity of disease [32, 33]. Antifibrotic treatment in patients with limited lung function impairment has been demonstrated to reduce FVC decline compared with placebo [28, 30, 31], and patients who progress on antifibrotic therapy still appear to benefit from continued therapy [34]. Furthermore, in a post-hoc analysis of data from the pooled ASCEND and CAPACITY population, pirfenidone showed similar efficacy in patients with more-preserved and less-preserved baseline lung function [27], a finding that has also been reported with nintedanib in a post-hoc subgroup analysis of data from the INPULSIS trials [29]. These data suggest that earlier treatment with antifibrotics may help to preserve lung function at higher levels if started in the early stages of the disease.

Goals for patient care may also have influenced treatment decisions. Overall, the three most important goals given by physicians were to prolong survival or reduce the risk of mortality, improve quality of life, and stabilize disease. However, the importance placed on these goals differed in treated and untreated patients, with improvement and/or stabilization in quality of life being more frequent for untreated patients (78%) than treated patients (68%). There may be a perception among physicians that antifibrotic treatment might have a detrimental effect on quality of life, possibly via the common side effects associated with treatment, and that the potential risks outweigh the benefit of treatment, particularly in patients with preserved lung function. Validating IPF-specific quality-of-life endpoints is still a work in progress and, so far, findings have been inconsistent with the treatment response (as measured with clinical endpoints) [35]. However, the available evidence from clinical trials suggests antifibrotic treatment results in statistically non-significant improvements in quality-of-life endpoints or has no net effect on these endpoints [15, 17, 36].

Access to IPF treatment differs in each country, for example because of reimbursement restrictions, and this may also have resulted in some differences in treatment practices. In Italy, patients must have a DLco > 35% and be < 80 years of age to be eligible for treatment reimbursement, while patients in the UK and in several regions in Spain must have an FVC < 80%. Interpretation of our data is limited because the survey did not specifically ask about access restrictions; however, the proportion of patients in the UK with ‘mild’ disease who were untreated was higher (51.4%) compared with countries without an upper FVC limit, such as France and Germany (41.1 and 40.4%, respectively). Overall, a greater proportion of the untreated population had an FVC > 80% compared with treated patients, both at diagnosis (17.9% vs 11.4%) and at last check up (13.1% vs 4.7%).

Both pirfenidone and nintedanib are associated with a number of adverse events, which may limit tolerability or result in treatment discontinuation due to potentially harmful events, e.g., rare elevations in liver enzymes [37, 38]. Patients may occasionally be prevented from taking specific antifibrotics due to contraindications [37, 38]. Although emphysema and lung cancer are not direct contraindications to antifibrotic treatment, untreated patients in our analysis were more likely to have these comorbidities. The results suggest that physicians are reluctant to treat patients with other lung diseases, perhaps due to a perception that these individuals may be more susceptible to adverse effects from treatment, or concerns about the benefit of treating fibrosis in individuals who may have other life-limiting disease. It should be noted, however, that patients with some degree of emphysema were included in the CAPACITY and INPULSIS trials [15, 17].

In Europe, pirfenidone is indicated for the treatment of ‘mild to moderate’ IPF [37], thereby excluding patients with ‘severe’ disease, often considered to be those with FVC < 50%. In fact, a minority of treated patients in our analysis had an FVC < 50%, and this proportion was similar in the untreated population. Other reasons for non-treatment could include patient choice, particularly in those with ‘mild disease’, or lack of adherence to treatment due to social or personal circumstances.

The focus of the current analysis was to investigate antifibrotic treatment patterns; however, in general, many patients in our analysis appeared to receive inadequate additional symptom management measures. Treatment guidelines recommend oxygen supplementation and other therapies for symptom control and management of comorbidities [2]; however, oxygen therapy and supportive treatments, such as anti-cough treatments, vaccines, etc., were used in only half of patients overall and in approximately a quarter of patients in the treated population. Supportive treatments for comorbidities, symptom control, or side-effect management may help with adherence to antifibrotic therapy and also improve patients’ perceptions of treatment. Furthermore, only 59/1783 patients (3%) overall received palliative care (46 patients were reported as having received palliative care only ± oxygen and a further 13 were reported as receiving no drug treatment and palliative care only ± oxygen). Amongst patients with IPF considered ‘severe’ by their doctor, only 10% were receiving palliative care. This is similar to previous findings, which highlighted poor or variable access and ineffective utilization of palliative care services, despite increasing evidence that access to palliative care services or having end-of-life discussions early in the course of IPF is desired by patients and can also improve quality of life, symptom control, and mood [21, 39–41]. Variable information regarding the disease and treatment, and access to other aspects of IPF management, such as supplementary oxygen, comorbidities, and palliative care, have been identified as unmet needs in the IPF Patient Charter; our observations support the findings of the Charter and suggest improvements in IPF awareness are still needed [21].

The conclusions from this study are limited by the nature of questionnaire-based research, which may have introduced bias. Furthermore, the quality of the data collected in this survey was reliant upon case notes recorded by physicians during patient consultations prior to their awareness of the survey. Physicians might not have reported on consecutive patients as directed, and may instead have selected cases that they considered representative of their medical decision making. The number of acute exacerbations resulting in hospitalization or an emergency room visit in the previous year (40–47%) was much higher than expected when compared with an annual acute exacerbation rate of 5–10% in the published literature [42–44], suggesting that these data are limited by the subjective acute exacerbation diagnoses made by individual physicians and the lack of specific criteria defining acute exacerbations in the survey. There is also no consensus on how to categorize disease severity or disease progression in IPF, and our analysis is limited by the subjective determination made by individual physicians as to whether disease was ‘mild’, ‘moderate’, or ‘severe’, or whether the patient had stable or worsening disease. We recognize that there is an unmet need for an objective severity staging system to capture the nature and progression of IPF. In particular, ‘mild’ is an inadequate description of a disease that can impair quality of life and undergo periods of acute exacerbation even in its early stages, and other classifications including ‘subclinical IPF’ may become more appropriate in the future. Finally, this analysis focused on five European countries (France, Germany, Italy, Spain, and the UK); therefore, the results may not be comparable in the rest of the world.

Treatment patterns in IPF will require further evaluation in future studies as more evidence is presented regarding available pharmacological treatments. The impact of early intervention and the potential for combining antifibrotics need further investigation [45, 46].

## Conclusion

In summary, this study highlights the high proportion of patients who are diagnosed with IPF, but do not receive antifibrotic treatment. The factors affecting treatment prescription in this analysis appear to involve diagnostic uncertainty and a lack of understanding around important features of both the disease and treatment as well as issues relating to treatment access. We acknowledge that a small proportion of patients will make an informed decision to not proceed with treatment. However, the adoption by physicians of a ‘watch and wait’ approach is of particular concern when evidence suggests immediate intervention can improve outcomes for patients with IPF. Increased education about IPF, in line with the European IPF Patient Charter, may help to empower patients to become more actively involved in treatment decisions and may improve treatment patterns in patients with IPF.

## Additional files

Additional file 1: Physician screening questionnaire. (DOCX 198 kb)

Additional file 2: Patient questionnaire. (DOCX 162 kb)

Additional file 3: Months until next consultation in the treated or untreated populations (excluding patients receiving only palliative care) for the pooled population and split by expert versus non-expert centers. (DOCX 14 kb)

## Abbreviations

6MWD: 6-min walk distance
CV: Cardiovascular
DLco: Carbon monoxide diffusing capacity of the lungs
FVC: Forced vital capacity
GERD: Gastroesophageal reflux disease
ILD: Interstitial lung disease
IPF: Idiopathic pulmonary fibrosis
MDT: Multidisciplinary team
MI: Myocardial infarction
NAC: N-acetylcysteine
SD: Standard deviation
