# Supplementary material for: Unmet needs in the treatment of idiopathic pulmonary fibrosis―insights from patient chart review in five European countries
Source: BMC Pulm Med. 2017 Sep 15;17:124. doi: 10.1186/s12890-017-0468-5 (PMC5602932; doi:10.1186/s12890-017-0468-5)
Supplement: Supplementary file 1 — Physician screening questionnaire. (DOCX 198 kb) [file 12890_2017_468_MOESM1_ESM.docx]

**Additional file 1** Physician screening questionnaire
