# Supplementary figures and images for: Unmet needs in the treatment of idiopathic pulmonary fibrosis―insights from patient chart review in five European countries
Source: BMC Pulm Med. 2017 Sep 15;17:124. doi: 10.1186/s12890-017-0468-5 (PMC5602932; doi:10.1186/s12890-017-0468-5)

**Additional file 2** Patient questionnaire


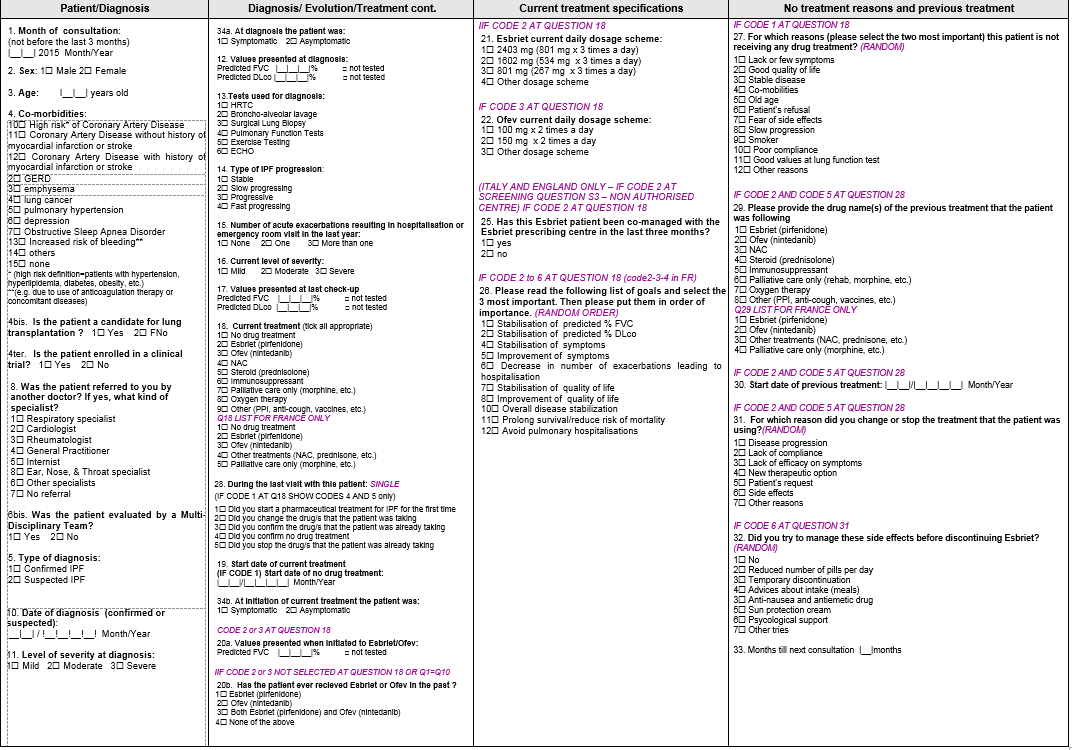

Supplement: Supplementary file 2 — Patient questionnaire. (DOCX 162 kb) [file 12890_2017_468_MOESM2_ESM.docx]
