# Supplementary material for: Unmet needs in the treatment of idiopathic pulmonary fibrosis―insights from patient chart review in five European countries
Source: BMC Pulm Med. 2017 Sep 15;17:124. doi: 10.1186/s12890-017-0468-5 (PMC5602932; doi:10.1186/s12890-017-0468-5)
Supplement: Supplementary file 3 — Months until next consultation in the treated or untreated populations (excluding patients receiving only palliative care) for the pooled population and split by expert versus non-expert centers. (DOCX 14 kb) [file 12890_2017_468_MOESM3_ESM.docx]

**Additional file 3** Months until next consultation in the treated or untreated populations (excluding patients receiving only palliative care) for the pooled population and split by expert versus non-expert centers

| **Months until next consultation, *n* (%)** | **Pooled population** | | **Treated** | | **Untreated** | |
| --- | --- | --- | --- | --- | --- | --- |
|  | **Treated  *N* = 828** | **Untreated  *N* = 909** | **Expert**  ***N* = 551** | **Non-expert**  ***N* = 277** | **Expert**  ***N* = 418** | **Non-expert**  ***N* = 491** |
| Less than 2 months | 142 (17.1) | 92 (10.1)^*^ | 103 (18.7) | 39 (14.1) | 47 (11.2) | 44 (9.0) |
| 2 months | 152 (18.4) | 141 (15.5) | 96 (17.4) | 56 (20.2) | 76 (18.2) | 65 (13.2) |
| 3 months | 409 (49.4) | 306 (33.7)^*^ | 249 (45.2) | 159 (57.4) | 141 (33.7.0) | 165 (33.6) |
| 4 months | 48 (5.8) | 109 (12.0)^*^ | 35 (6.4) | 14 (5.1) | 45 (10.8) | 64 (13.0) |
| 5 months | 11 (1.3) | 19 (2.1) | 8 (1.5) | 2 (0.7) | 10 (2.4) | 9 (1.8) |
| 6 months | 54 (6.5) | 199 (21.9)^*^ | 47 (8.5) | 7 (2.5) | 81 (19.4) | 118 (24.0) |
| >6 months | 12 (1.4) | 43 (4.7)^*^ | 12 (2.2) | - | 18 (4.3) | 26 (5.3) |

*p* values represent treated population versus untreated population; ^*^*p* ≤ 0.01
